# Factors Affecting Cerebral Oxygenation in Hemodialysis Patients: Cerebral Oxygenation Associates with pH, Hemodialysis Duration, Serum Albumin Concentration, and Diabetes Mellitus

**DOI:** 10.1371/journal.pone.0117474

**Published:** 2015-02-23

**Authors:** Kiyonori Ito, Susumu Ookawara, Yuichiro Ueda, Sawako Goto, Haruhisa Miyazawa, Hodaka Yamada, Taisuke Kitano, Mitsunobu Shindo, Yoshio Kaku, Keiji Hirai, Masashi Yoshida, Taro Hoshino, Aoi Nabata, Honami Mori, Izumi Yoshida, Masafumi Kakei, Kaoru Tabei

**Affiliations:** 1 Division of Nephrology, Department of Integrated Medicine, Saitama Medical Center, Jichi Medical University, Saitama, Japan; 2 Department of Internal Medicine, Nishikawa Town Hospital, Yamagata, Japan; 3 Division of Endocrinology and Metabolism, Department of Integrated Medicine Saitama Medical Center, Jichi Medical University, Saitama, Japan; National University of Singapore, SINGAPORE

## Abstract

**Background:**

Patients undergoing hemodialysis (HD) often develop cerebral disease complications. Furthermore, cerebral regional saturation of oxygen (rSO_2_) was previously reported to be significantly lower in HD patients than in healthy subjects. We aimed to identify the factors affecting the cerebral rSO_2_ in HD patients.

**Methods:**

Fifty-four HD patients (38 men and 16 women; mean age, 67.7 ± 1.2 years, HD duration, 6.5 ± 1.9 years) were recruited. Cerebral rSO_2_ was monitored at the forehead before HD using an INVOS 5100C (Covidien Japan, Tokyo, Japan).

**Results:**

The rSO_2_ levels were significantly lower in HD patients compared with healthy controls (49.5 ± 1.7% vs. 68.9 ± 1.6%, p <0.001). Multiple regression analysis showed that cerebral rSO_2_ independently associated with pH (standardized coefficient: -0.35), HD duration (standardized coefficient: -0.33), and serum albumin concentration (standardized coefficient: 0.28). Furthermore, the rSO_2_ was significantly lower in HD patients with diabetes mellitus (DM), compared with patients without DM (46.8 ± 1.7% vs. 52.1 ± 1.8%, p <0.05).

**Conclusions:**

In HD patients, cerebral rSO_2_ was affected by multiple factors, including pH, HD duration, and serum albumin concentration. Furthermore, this is the first report describing significantly lower levels of rSO_2_ in HD patients with DM than in those without DM.

## Introduction

Central nervous system (CNS) dysfunction, such as uremic encephalopathy, cognitive impairment, and dementia, is a frequent complication of patients undergoing hemodialysis (HD). [1] Cerebrovascular accident (CVA) was described as the fourth leading cause of death in HD patients according to the annual report of the Japanese Society for Dialysis Therapy in 2011. [2] Magnetic resonance imaging (MRI) is a useful tool for detecting morphological changes in the brain and therefore evaluating CVA; in addition, silent cerebral infarction detected by MRI has been found to associate with the severity of cognitive impairment in HD patients. [3] However, imaging methods like MRI and computed tomography can only provide information about organic lesions in the brain, and cannot evaluate the functional status such as cerebral blood flow and cerebral oxygenation. Recently, near-infrared spectroscopy (NIRS) has been used as a tool to measure the regional saturation of oxygen (rSO_2_), a marker of tissue oxygenation, at the frontal cerebral cortex in a variety of clinical situations, and has shown the change of critical balance between arterial oxygen delivery and cerebral oxygen consumption. [4–7] Cerebral rSO_2_ was reported to be significantly lower in HD patients than in healthy controls. [1,8] Few reports, however, have examined the relationship between cerebral oxygenation in HD patients and clinical parameters. Therefore, in this study, we aimed to elucidate the clinical factors influencing cerebral rSO_2_ in HD patients.

## Methods

In this study, we included HD patients who met the following criteria: (1) patients with end-stage renal disease receiving intermittent HD and (2) patients with unimpaired consciousness. The exclusion criteria were: (1) coexisting disease including chronic obstructive pulmonary disease, apparent neurological disorder, and chronic hypotension (defined as systolic blood pressure <100 mmHg), and (2) history of cerebrovascular disease and dementia. Not all patients enrolled in this study underwent imaging examinations such as computed tomography and MRI for detecting cerebral ischemia, carotid artery stenosis or aortic stenosis. Therefore, we cannot completely exclude the existence of ischemic conditions in each patient. However, we excluded HD patients with apparent neurological disorder, history of cerebrovascular disease, and dementia; therefore, it could be considered that cerebral ischemia, carotid artery stenosis or aortic stenosis had no clinical effect in the HD patients enrolled in our study. Fifty four HD patients were recruited (38 men and 16 women; mean age, 67.7 ± 1.2 years, HD duration, 6.5 ± 1.9 years). The causes of chronic renal failure were diabetes mellitus (DM, 27 patients), chronic glomerulonephritis (14 patients), nephrosclerosis (4 patients), polycystic kidney disease (4 patients), and other (5 patients). Each patient received maintenance HD 2 or 3 times a week, and the duration of the HD sessions was 3 or 4 h. The patients’ general characteristics are summarized in Table 1. All participants signed informed consent to participate in this study. This study was approved by the Institutional Review Board of Saitama Medical Center, Jichi Medical University, Japan (No. RIN13-39), and Nishikawa Town Hospital, Japan (No. 1/4/2013), and conforms to the provisions of the Declaration of Helsinki (as revised in Tokyo in 2004). In addition, 28 healthy volunteers (18 men and 10 women, mean age, 43.4 ± 3.6 years) were recruited as the control group.

**Table 1:** Patients Characteristics and the correlation between cerebral rSO_2_ and clinical parameters.

	mean ± SE	p value	r
Total number pf patients (male/female)	54 (38/16)		
Age (years)	67.7 ± 1.2	NS	
Disease			
diabetes mellitus	27		
chronic glomerulonephritis	14		
nephrosclerosis	4		
polycystic kidney disease	4		
others	5		
HD duration (years)	6.6 ± 0.9	<0.01	-0.35
Weight gain (%)	3.4 ± 0.2	NS	
Systolic blood pressure (mmHg)	139.7 ± 2.8	NS	
Diastolic blood pressure (mmHg)	73.2 ± 1.8	NS	
pH	7.38 ± 0.0	<0.01	-0.42
pCO2 (mmHg)	37.6 ± 0.6	NS	
pO_2_ (mmHg)	81.9 ± 2.2	NS	
HCO3^-^ (mEq/L)	21.6 ± 0.3	NS	
Sat O2 (%)	94.8 ± 0.6	NS	
Hb (g/dL)	9.9 ± 0.2	<0.01	0.44
Arterial O2 content (mL/dL)	12.8 ± 0.2	<0.01	0.40
ESAs dose (U/week)	5375 ± 510	NS	
BUN (mg/dL)	52.1 ± 2.2	<0.05	0.29
Cr (mg/dL)	8.5 ± 0.3	NS	
Na (mEq/L)	137.1 ± 0.5	<0.01	0.45
K (mEq/L)	4.5 ± 0.1	<0.05	0.28
Ca (mg/dL)	8.9 ± 0.1	<0.05	-0.28
P (mg/dL)	4.7 ± 0.2	<0.01	0.35
Total protein (g/dL)	6.2 ± 0.1	NS	
Serum albumin (g/dL)	3.3 ± 0.1	<0.01	0.41
Serum osmolarity (mosm/kg-H_2_O)	301.5 ± 1.4	<0.05	-0.33
Plasma glucose (mg/dL)	156.6 ± 8.5	<0.01	0.36
HbA1c (%)	5.9 ± 0.2	NS	
C-reactive protein (mg/dL)	2.2 ± 0.6	<0.01	-0.36

### Monitoring of cerebral oxygenation and clinical laboratory measurement

Cerebral rSO_2_ was monitored at the forehead with an INVOS 5100C saturation monitor (Covidien Japan, Tokyo, Japan), which utilizes NIRS technology. This instrument uses a light-emitting diode, which transmits near-infrared light at 2 wavelengths (735 and 810 nm), and 2 silicon photodiodes which act as light detectors; results are read as a single numerical value that represents the rSO_2_ [9,10]. All data obtained by this instrument were immediately and automatically stored in sequence. Interobserver variance for this instrument, that is, reproducibility of the rSO_2_ measurement, is acceptable as previously reported. Therefore, rSO_2_ is considered reliable when estimating the actual cerebral oxygenation. [11]

Prior to HD, the recruited patients rested in the supine position for at least 10 min in order to reduce the influence of postural change. An rSO_2_ measurement sensor was attached to the patient’s forehead for measurement in the resting state. Thereafter, rSO_2_ was measured for 5 min before HD, and we evaluated the mean rSO_2_ for 5 min, as a marker of cerebral oxygenation, in each patient. Blood samples were obtained from each patient under room air. It was previously reported that samples obtained from the radial artery or those from an arterial line at the arteriovenous fistula presented similar values when evaluating the parameters of oxygen status, including pH, oxygen pressure (pO_2_: mmHg), and oxygen saturation (SpO2: %). [12] Therefore, prior to HD we obtained all blood samples, including blood gas analysis, from the arterial site of arteriovenous fistulae in each patient.

Arterial O2 content (CaO_2_) and serum osmolality (sOsm) were calculated using the following equations:

$${\text{CaO}}_{2}(\text{mL/dL})=1.34\;\times \;\text{Hb}\;\times \;\text{SpO}2\;÷\;100\;+\;(0.0031\;\times \;{\text{pO}}_{2})$$

[13]

$$\text{sOsm}(\text{mosm/kg}-{\text{H}}_{2}\text{O})=(2\;\times \;\text{Na})+\;\text{PG}\;÷\;18\;+\;\text{BUN}\;÷\;2.8$$

[14] where Hb represents the hemoglobin concentration (g/dL). Na represents the serum sodium concentration (mEq/L), PG represents the plasma glucose level (mg/dL), and BUN represents the blood urea nitrogen concentration (mg/dL).

Erythropoiesis-stimulating agents (ESAs) were administered for the treatment of renal anemia, and calculations of the optimum ESA dose (U/week) were based on a method reported previously, [15] where a ratio of 1:200 was used to convert the dose for long-acting ESAs, including darbepoetin-α and continuous erythropoietin receptor activator, into a short-acting recombinant human erythropoietin equivalent dose for each patient. [15] The rSO_2_ in healthy controls was measured for at least 5 min in the supine position in a manner similar to that in HD patients.

## Analysis

Data were expressed as mean ± standard error (SE). The Student’s t-test for non-paired values was used for comparing 2 groups, and Mann–Whitney U test was used for comparison of nonparametric variables between 2 groups. Correlations between 2 groups were evaluated by Pearson’s correlation coefficient and linear regression analysis. Multiple regression analysis was performed using parameters that showed a significant correlation with cerebral rSO_2_. A difference of p <0.05 was considered significant.

## Results

**Fig 1:**
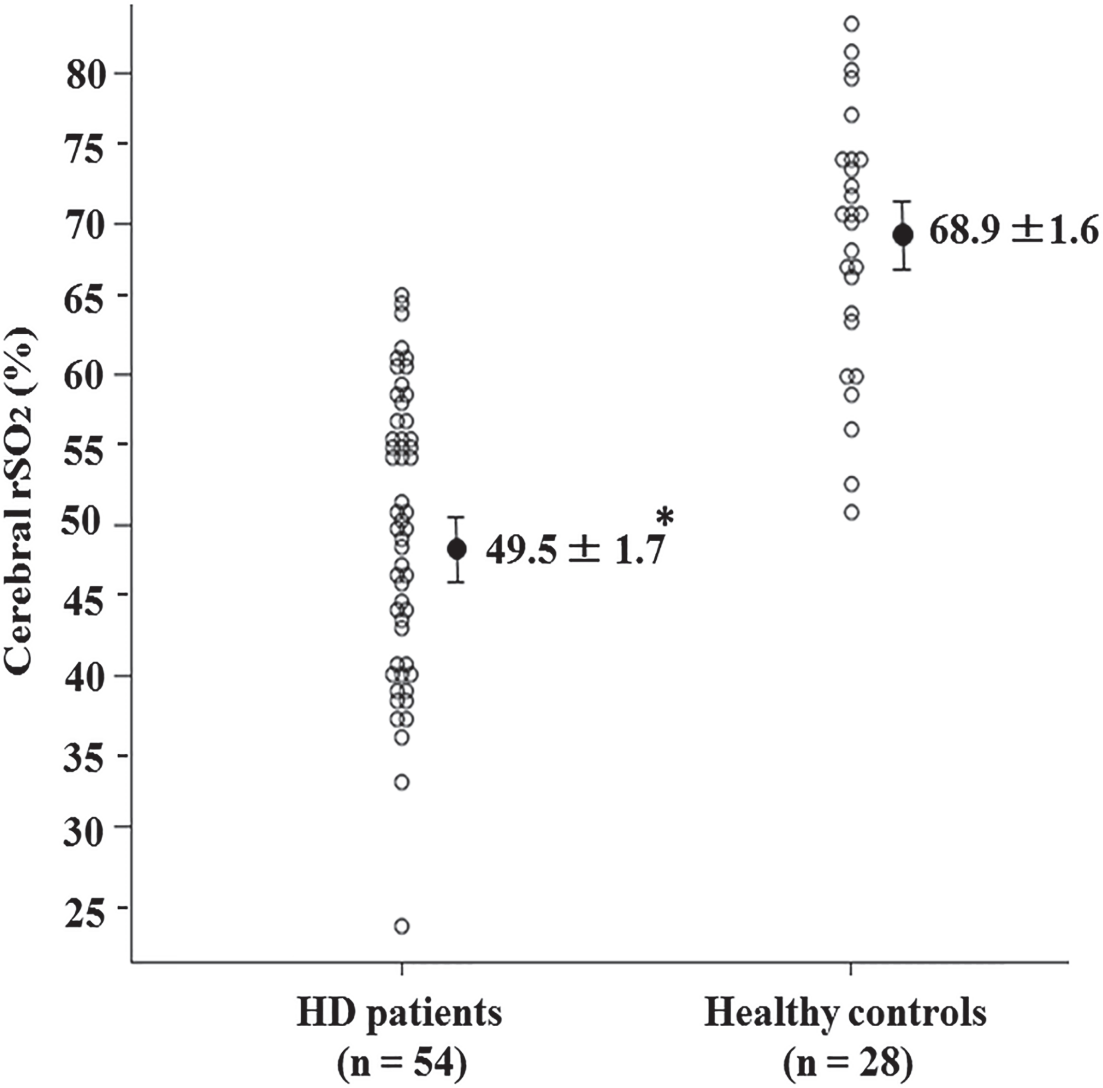
Comparison between cerebral rSO_2_ in hemodialysis patients and healthy controls.

Cerebral rSO_2_ at rest in HD patients was compared with that in healthy controls, and there was a significant difference between the 2 groups (HD patients: 49.5 ± 1.7%, healthy controls: 68.9 ± 1.6%, p < 0.001) (Fig. 1). Recently, cerebral rSO_2_ was reported to be significantly lower in HD patients than in healthy controls [1, 8], and our results are consistent with these reports.

Table 1 shows patients’ characteristics, and correlations between the cerebral rSO_2_ and clinical parameters. Cerebral rSO_2_ showed significant positive correlations with CaO_2_, hemoglobin (Hb) level, serum sodium concentration, serum potassium concentration, serum inorganic phosphate concentration, serum albumin concentration, and plasma glucose level. A simple linear regression analysis revealed that cerebral rSO_2_ was negatively correlated with pH, serum calcium concentration, sOsm, HD duration, and C-reactive protein.

**Fig 2:**
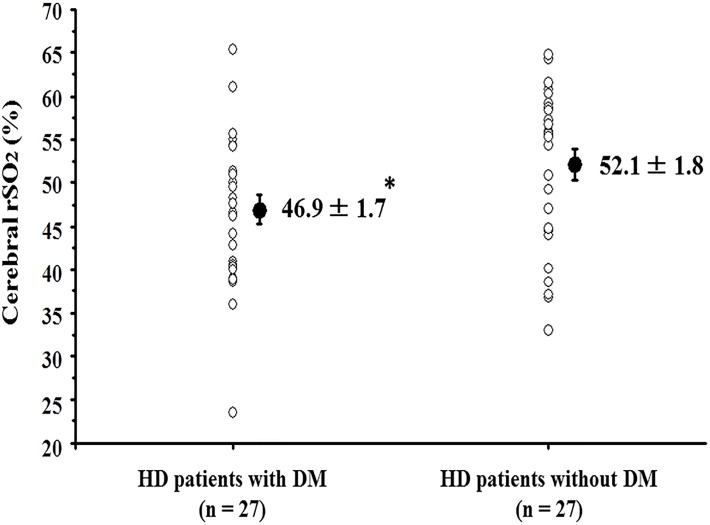
Comparison of cerebral rSO_2_ in hemodialysis patients (HD) with and without diabetes mellitus (DM). * < 0.05 at HD patients with DM vs. those without DM.

We performed a multivariate linear regression analysis using variables that showed a significant correlation with the cerebral rSO_2_ in a simple linear regression analysis (Table 2). The multivariate regression analysis found that the cerebral rSO_2_ was independently associated with pH (standardized coefficient: -0.35), HD duration (standardized coefficient: -0.33), and serum albumin concentration (standardized coefficient: 0.28). On the other hand, the cerebral rSO_2_ was independent of Hb and CaO_2_. We also evaluated the influence of DM on the cerebral rSO_2_ values (Table 3). The cerebral rSO_2_ was significantly lower in patients with DM than in those without DM (46.8 ± 1.7% vs. 52.1 ± 1.8, p < 0.05) (Fig. 2). In addition to the difference in cerebral rSO_2_, there were significant differences in serum sodium concentration, plasma glucose level, and HbA1c levels between the 2 groups.

**Table 2:** Multivariate linear regression analysis: Independent factors of cerebral rSO_2_ in hemodialysis patients.

Variables	Coefficient	Standardized coefficient	p
pH	-62.5	-0.35	0.012
HD duration	-0.47	-0.33	0.006
Serum albumin	4.12	0.28	0.041

**Table 3:** Different clinical parameters for hemodialysis patients with and without diabetes mellitus.

	with DM	without DM	p
rSO_2_ (%)	46.9 ± 1.7	52.1 ± 1.8	<0.05
Age (years)	66.3 ± 1.4	69.1 ± 2.0	NS
Male/Female	18 / 9	20 / 7	NS
HD duration (years)	7.7 ± 1.4	5.4 ± 1.1	NS
Weight gain (%)	3.6 ± 0.4	3.3 ± 0.3	NS
Systolic blood pressure (mmHg)	144.8 ± 3.7	134.6 ± 4.1	NS
Diastolic blood pressure (mmHg)	74.0 ± 2.6	72.4 ± 2.3	NS
pH	7.37 ± 0.01	7.38 ± 0.01	NS
pCO2 (mmHg)	38.3 ± 0.6	36.9 ± 1.1	NS
pO_2_ (mmHg)	80.1 ± 3.2	83.7 ± 2.9	NS
HCO3^-^ (mEq/L)	21.8 ± 0.4	21.3 ± 0.5	NS
Sat O2 (%)	94.5 ± 0.6	95.1 ± 1.0	NS
Hb (g/dL)	10.2 ± 0.2	9.6 ± 0.2	NS
Arterial O2 content (mL/dL)	13.1 ± 0.3	12.5 ± 0.3	NS
ESAs dose (U/week)	5287 ± 797	5463 ± 651	NS
BUN (mg/dL)	48.1 ± 3.0	56.0 ± 3.2	NS
Na (mEq/L)	136.1 ± 0.6	138.1 ± 0.7	NS
K (mEq/L)	4.4 ± 0.1	4.5 ± 0.2	NS
Ca (mg/dL)	8.9 ± 0.1	9.0 ± 0.1	NS
P (mg/dL)	4.7 ± 0.3	4.8 ± 0.2	NS
Serum albumin (g/dL)	3.3 ± 0.1	3.2 ± 0.1	NS
Serum osmolarity (mosm/kg-H_2_O)	299.6 ± 2.0	303.5 ± 2.0	NS
Plasma glucose (mg/dL)	181.9 ± 14.5	131.5 ± 6.2	<0.01
HbA1c (%)	6.6 ± 0.2	5.1 ± 0.1	<0.01

## Discussion

Regional saturation of oxygen (rSO_2_) is widely used for monitoring cerebral function during cerebral surgery, as rSO_2_ measured using NIRS can provide accurate yet non-invasive information on cerebral oxygen saturation, and can be easily performed in the clinical setting. [1, 4–11] Recently, cerebral rSO_2_ was reported to be significantly lower in HD patients than in healthy controls. [1,8] The reasons for this however, remain uncertain, and the factors affecting the deterioration of cerebral rSO_2_ in HD patients have not been determined. In this study, we identified modifiable factors, including pH, HD duration, and serum albumin concentration, as being independently associated with cerebral rSO_2_; we also demonstrated that cerebral rSO_2_ was significantly lower in HD patients with DM than in those without DM.

Among the modifiable factors identified as being independently associated with cerebral rSO_2_, pH was the factor most strongly affecting cerebral rSO_2_ in HD patients. A decrease of extracellular pH, even without partial pressure of carbon dioxide (pCO2) increase, was previously reported to induce a dilation of the cerebral artery, [16] and therefore, regional cerebral blood flow (rCBF) would increase in response to the decrease in pH. Thus, in the brain, it is possible that cerebral rSO_2_ increases via the increase of arterial oxygen delivery accompanying rCBF increase induced by pH decrease. This mechanism could explain why cerebral rSO_2_ shows an inverse relationship with pH change. Furthermore, changes in cerebral rSO_2_ were recently shown to be independently and negatively associated with changes in pH in patients undergoing liver transplantation, [17] and our results, which indicated an inverse relationship between cerebral rSO_2_ and pH, were consistent with this report. Thus far, however, the change of rSO_2_ affected by pH remains unclear, so further examination would be required regarding the association between cerebral rSO_2_ change and pH.

Serum albumin concentration also showed a significant positive correlation with cerebral rSO_2_. Serum albumin concentration was previously reported to be a prognostic marker of survival in HD patients, similar to nutritional status. [18,19] The decrease in its concentration has often been observed in patients with protein-energy malnutrition, leading to prognostic aggravation in HD patients. In general, serum albumin concentration contributes to the formation of colloid osmotic pressure in vessels and associates with body-fluid movement, mainly between the vessels and the interstitium. In addition, serum albumin concentration was recently shown to positively correlate with the regional cerebral blood flow in patients with liver cirrhosis. [20] Based on our results, we propose that an increase in serum albumin concentration might lead to increased rCBF and improved cerebral oxygenation, which can then be measured as cerebral rSO_2_. Therefore, serum albumin concentration would appear to associate not only with nutritional status and prognosis, but also with cerebral oxygenation in HD patients, although its precise mechanism remains uncertain.

**Fig 3:**
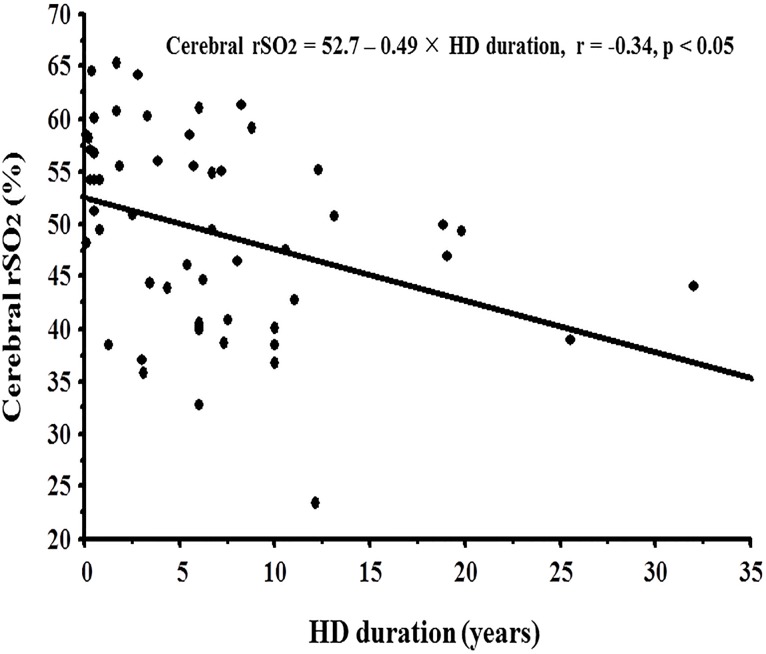
Correlation between hemodialysis duration and rSO_2_.

Furthermore, in this study, the cerebral rSO_2_ was negatively affected by HD duration, and the annual rSO_2_ decline in HD patients was predicted to be -0.49%/year by simple linear regression analysis (Fig. 3). It was previously reported that, in HD patients, rCBF to the frontal cortex decreased with an increase in HD duration, resulting in white matter lesions. [21] As the cerebral rSO_2_ mainly indicates the condition of rCBF, the negative impact of HD duration on cerebral rSO_2_ might be due to a decrease in the rCBF.

On the other hand, although CaO_2_ and Hb levels significantly correlated with cerebral rSO_2_ in a simple linear regression analysis, these associations disappeared upon multivariate linear regression analysis. Hemoglobin is an important factor in oxygen supply to the peripheral tissues and organs, including the brain; CaO_2_ is a marker for oxygen supply. Thus, cerebral rSO_2_ might be expected to show a strong correlation with Hb and CaO_2_ levels; however, no significant correlations were observed in the present study. Positron emission tomography analysis has revealed an association between the rCBF and Hb levels in HD patients. [22] In this report, regional cerebral blood flow decreased significantly, and oxygen metabolism was disrupted despite the increase in Hb levels. Decreased blood cell deformability and increased plasma viscosity resulted in decreased erythrocyte velocity in the cerebral capillaries, leading to an increase in Hb levels. Furthermore, in HD patients, CaO_2_ was reported to be the most important determinant of inter-individual middle cerebral artery (MCA) blood flow velocity variance and variation; in addition, an increase in CaO_2_ could induce the decrease of MCA blood flow velocity via vasoconstrictions in small intracerebral vessels to maintain oxygen delivery to the brain. [23,24] Indeed, in an experimental study, cerebral O2 transport (CBF × CaO_2_) was regulated at constant levels, independently of alterations in Hb levels and CaO_2_ values. [25] Therefore, it is unlikely that oxygen metabolism including rSO_2_ could directly associate with Hb and CaO_2_ levels. Furthermore, ESA administration was reported to reduce the development of brain edema, and preserve the local brain oxygen saturation and brain tissue oxygenation after traumatic brain injury. [26,27] However, there was no association between cerebral rSO_2_ values and ESA doses in HD patients.

This study included the results obtained from 1 patient with DM, which showed an extremely low cerebral rSO_2_ value. Thus, we analyzed the cerebral rSO_2_ values using the Grubbs-Smirnov rejection test to clarify whether the extremely low rSO_2_ value should be excluded. The test results were not significant; therefore, based on the data obtained in this study, a comparison of cerebral rSO_2_ values was performed for cohorts of HD patients with, and without, DM. The results, which showed significant differences in cerebral rSO_2_ in HD patients with DM compared with those without DM, are interesting, because it was recently suggested that dementia and cognitive impairment were related to DM and chronic kidney disease in patients, including those that underwent HD. [28,29] In particular, DM has previously been reported to be a significant risk factor for Alzheimer’s disease and vascular dementia. [30] In addition, an increase in fasting plasma glucose was associated with functional impairment of regional cerebral perfusion; moreover, an improvement in glycemic control led to a reduction in cerebral perfusion deficits. [31] In this report, impaired regional cerebral perfusion could be induced by hyperglycemia-induced endothelial dysfunction. Furthermore, dynamic cerebral autoregulation was reported to be impaired even during an early phase in type 2 DM patients. [32] In this study, plasma glucose and HbA1c levels were significantly higher in patients with DM than in those without DM; therefore, the significant decrease in cerebral rSO_2_ with DM might be induced by the dysregulation of regional cerebral perfusion due to hyperglycemia-induced endothelial dysfunction. In the present study, there was a difference of ~5% in the cerebral rSO_2_ between HD patients with and without DM, and this value corresponds to cerebral rSO_2_ decline for nearly 10 years, at an annual rate of decline of -0.49%/year for rSO_2_ in HD patients; calculations for the rate of decline were based on the negative correlation between cerebral rSO_2_ and HD duration. Therefore, these results might explain the mechanisms underlying the frequent occurrence of cerebral complications such as dementia and cognitive impairment in HD patients with DM.

This study faced the limitation of a relatively small sample size; therefore, further study is required to fully elucidate the correlation of cerebral rSO_2_ with various clinical parameters.

In conclusion, cerebral rSO_2_ was affected by multiple factors in HD patients, including pH, HD duration, and serum albumin concentration. Furthermore, rSO_2_ was significantly lower in HD patients with DM than in those without DM.
